# The Dickman Impulsivity Inventory: Validation and measurement invariance among Portuguese young adults

**DOI:** 10.1371/journal.pone.0260621

**Published:** 2021-12-02

**Authors:** Pedro Pechorro, Rebecca Revilla, Miguel Resende, Rui Abrunhosa Gonçalves, Cristina Nunes, Melissa A. Cyders

**Affiliations:** 1 School of Psychology, University of Minho, Campus de Gualtar, Braga, Portugal; 2 Department of Psychology, University of Alabama, Tuscaloosa, Alabama, United States of America; 3 Psychology Research Centre (CIP), University of Algarve, Campus de Gambelas, Faro, Portugal; 4 Department of Psychology, School of Science, Indiana University Purdue University Indianapolis, Indianapolis, Indiana, United States of America; University of Queensland, AUSTRALIA

## Abstract

The Dickman Impulsivity Inventory (DII) measures impulsive personality related to both negative and positive behaviors and characteristics. The main aim of the present study was to examine the psychometric properties of the DII among a Southern-European sample of Portuguese young adults. Our convenience sample (*N* = 429, *M* = 22.11 years, *SD* = 3.35, range = 18–42), composed of women (*n* = 237, *M* = 22.08 years, *SD* = 3.35, age range = 18–42) and men (*n* = 192, *M* = 22.14 years, *SD* = 3.34, range = 18–35), was collected from a university context. The two-factor latent structure of the DII composed of functional and dysfunctional impulsivity was supported, although three items had to be removed due to low standardized loadings, and strong cross-gender measurement invariance was established. Our analyses of the DII also provided evidence of criterion-related validity, known-groups validity, and internal consistency/reliability. Our findings support the use of the DII among Portuguese young adults.

## Introduction

The personality trait of impulsivity is related to both harmful risk-taking behaviors and positive characteristics, such as fast information processing [1–3]. Through various studies on impulsivity and cognitive functioning, Dickman [4, 5] found impulsivity can be separated into two traits: dysfunctional impulsivity, which relates to negative outcomes, and functional impulsivity, which relates to positive outcomes. Although both constructs are related to individuals acting quickly with little thought, they have a small correlation with each other and individuals high in these constructs appear to have different personality traits, cognitive functioning, and behavioral outcomes [5–7]. Dysfunctional impulsivity is more strongly associated with disorderliness, a tendency to make decisions without wanting additional information, disliking work that requires cautiousness, and disliking planning ahead [5]. Functional impulsivity is more strongly associated with adventurousness and enthusiasm [5]. Overall, Dickman suggests dysfunctional impulsivity and functional impulsivity both represent a tendency to perform rapidly and inaccurately. Individuals high in functional impulsivity will perform in this manner when it is optimal for achieving a beneficial outcome while individuals high on dysfunctional impulsivity may struggle to regulate themselves in this way [8].

The Dickman Impulsivity Inventory (DII) was created to measure both dysfunctional and functional impulsivity constructs [5]. Evidence of validity for the DII was originally provided in samples of English-speaking undergraduate students and showed two factors: A Dysfunctional Impulsivity factor, consisting of 12 items that loaded above .30 and a Cronbach’s alpha of .86, and a Functional Impulsivity factor, consisting of 11 items that loaded above .30 and a Cronbach’s alpha of .83. The DII has been adapted and tested in various languages, including French, Dutch, Spanish, Italian, Chinese, and Brazilian-Portuguese [9–14]. These studies confirmed that the expected two-factor model obtained a better fit when compared to other possible models and found a low, significant correlation between the two factors, although the Italian [12] and Brazilian-Portuguese [14] versions had to exclude several items to fit a two-factor structure. In the Italian version, Colledani [12] removed items 8 and 20 (“I have often missed out on opportunities because I couldn’t make up my mind fast enough”; “People have admired me because I can think quickly”) from the Functional Impulsivity factor and items 4, 17, and 23 from the Dysfunctional Impulsivity factor (“I enjoy working out problems slowly and carefully”; “Many times the plans I make don’t work out be- cause I haven’t gone over them carefully enough in advance”; “I rarely get involved in projects without first considering the potential problems”) after items either loaded on both factors or items had low factor loadings. It was not specified which items fell into each category. In the Brazilian-Portuguese version, Gomes and colleagues [14] removed items 4, 7, 9, and 10 (“I enjoy working out problems slowly and carefully”; “I often make up my mind without taking the time to consider the situation from all angles”; “I often say and do things without considering the consequences”; “I frequently make appointments without thinking about whether I will be able to keep them”) for loading on the opposite factor and removed item 15 (“I like to take part in really fast-paced conversations, where you don’t have much time to think before you speak”) for having a low factor loading on both factors. Similar problems were reported in the French version [9] with item 9 (“Most of the time, I can put my thoughts into words very rapidly”), the Dutch [10] version with items 4, 8, and 23 (“I enjoy working out problems slowly and carefully”; “I have often missed out on opportunities because I couldn’t make up my mind fast enough”, “I rarely get involved in projects without considering the potential problems”), and the Spanish [11] version with item 4.

Several of these studies evaluated the DII across gender and found the factors to be similar across men and women with a few exceptions. Specifically, Colledani [12] found the correlation between functional and dysfunctional impulsivity was higher in women than in men and the functional impulsivity factor mean was higher in men than in women. These differences align with results reported in a meta-analysis on gender differences in impulsivity [15]. Claes and colleagues [10] found men reported significantly more functional and dysfunctional impulsivity than women. Caci and colleagues [9] found differences across gender on items 2, 8, 18, 20, and 21 and suggested the DII may not be understood in the same way across gender.

Studies that have provided validity evidence of the DII often evaluate the two constructs in relation to the Impulsiveness-Venturesomeness-Empathy questionnaire [10, 16]. Although most studies focus solely on the Impulsiveness and Venturesomeness components of the questionnaire, Colledani [12], found the empathy component negatively relates to Functional Impulsivity and may also be useful for providing evidence of validity. Other constructs that can relate to impulsivity include narcissism, psychopathy, aggression, and substance use [17–19]. Specifically, Functional Impulsivity is more related to narcissism, whereas Dysfunctional Impulsivity is more related to psychopathy [17]. Dysfunctional Impulsivity is also related to patterns of binge drinking, physical aggression, and verbal aggression while Functional Impulsivity is not [20, 21]. Thus, each of these constructs can provide validity evidence.

Although the DII is the most widely used and adapted measure of Functional and Dysfunctional Impulsivity, it has not been adapted for use in Portugal. It is essential to adapt the DII for use in Portugal to be able to compare these constructs across studies. Thus, the goal of this study is to translate the DII into European Portuguese (Pt-Pt), evaluate the psychometric properties of this version, and test for gender measurement invariance among a sample of Portuguese university students. We have seven aims to achieve this goal. The first aim is to test the factor structure of the DII using confirmatory factor analysis. The second aim is to test measurement invariance between men and women by examining how the psychometric properties on the scale may differ by group. The third aim is to provide evidence of criterion-related validity by identifying the relationships among DII functional impulsivity factor, DII dysfunctional impulsivity factor, and alcohol and drug use, empathy, reactive-proactive aggression, and triarchic psychopathic traits. The fourth aim is to provide evidence of known-groups validity between men and women. Lastly, we aim to provide evidence of internal consistency of the scale across men and women using item-total correlations, mean inter-item correlations, Cronbach´s alpha, and omega coefficients.

We predict that the DII will show: 1) the presumed latent 2-factor structure; 2) measurement invariance; 3) evidence of criterion-related validity (e.g., dysfunctional impulsivity associated with measures of alcohol and drug use, basic empathy, reactive-proactive aggression and triarchic psychopathic traits); 4) evidence of known-groups validity (e.g., men versus women groups); and 5) evidence of adequate internal consistency measured by item-total correlations, mean inter-item correlations, Cronbach´s alpha, and omega coefficients. It is critical to provide evidence of the validity and reliability of the translated DII scale to determine if it can be used with Portuguese young adult populations. Also, providing evidence of measurement invariance will allow comparisons on the DII to be made between groups of men and women.

## Method

### Participants

A sample of 429 university students (*M* = 22.11 years, *SD* = 3.35, range = 18–42) voluntarily participated in this study. The sample was subdivided into women (*n* = 237, *M* = 22.08 years, *SD* = 3.35, range = 18–42) and men (*n* = 192, *M* = 22.14 years, *SD* = 3.34, range = 18–35 years). The gender groups’ mean ages were not significantly different (*F* = .034, *p* = .85). This convenience sample was collected from the University of Minho at Braga (Gualtar campus), a state university from the northern region of Portugal. These university students were mostly Portuguese nationals (95.6%) and Brazilian nationals (3.3%) majoring in the social sciences (51%) and sciences and technologies areas (39.9%).

### Procedures

In the initial phase of the DII adaptation process, the authors of the present study followed a common translation/back-translation procedure [22]. First and third authors translated the measure into the European Portuguese (Pt-Pt) language spoken in Portugal. A native English-speaking translator with considerable experience completed the back-translation. The translators made small adjustments so the English version and the Portuguese version of the DII would show no relevant differences (e.g., in the translation of item 21, the initial translation of weigh, *peso* was replaced by *penso* to convey a culturally appropriate meaning). Following this step, the translation was tested with individual participants, as well as among small groups of participants, to ensure that it could be easily comprehended. This step is important when translating a test because it indicates whether the translators/back-translators fully conveyed the meaning of the corresponding terms and vocabularies in the respective languages [23]. The final Portuguese translation of the DII is presented in S1 Appendix.

A four-point response format was adopted in the present study because there is evidence that adapting psychometric measures from the dichotomous format to the polytomous format usually improves evidence of validity and reliability [24]. Some previous studies evaluating validity of the DII also adopted a polytomous format [14].

The Ethics committee of the School of Psychology–University of Minho authorized the assessment of the participants in person and online. After learning about the present study, participants were asked to voluntarily and anonymously complete questionnaires during their university classes. All participants signed a written informed consent. No financial compensation or other form of compensation was given for participating.

All procedures followed were in accordance with the ethical standards of the responsible committee on human experimentation (institutional and national) and with the Helsinki Declaration of 1975, as revised in 2000.

### Measures

*Dickman Impulsivity Inventory* (DII) [5]. This is a 23-item self-report measure of impulsivity that consists of two scales: functional impulsivity (11 items; e.g., “Most of the time, I can put my thoughts into words very rapidly.”, “People have admired me because I can think quickly.”), and dysfunctional impulsivity (12 items; e.g., “I will often say whatever comes into my head without thinking first.”, “I often get into trouble because I don’t think before I act.”). The original version of the DII used a dichotomous response format, but in the present study we adopted a four-point format (0 = *Totally false*, 1 = *In part false*, 2 = *In part true*, 3 = *Totally true*). Items are summed to create each scale. Higher scores indicate higher levels of functional and dysfunctional impulsivity characteristics.

*Basic Empathy Scale* (BES) [25]. This is a 20-item self-report measure of empathy that consists of two scales: affective empathy (11 items; e.g., “I don’t become sad when I see other people crying.”, “Other people’s feelings don’t bother me at all.”) and cognitive empathy (9 items; e.g., “I can usually workout when people are cheerful.”, “I can usually realize quickly when a friend is angry.”). Each item is scored on an ordinal 5-point Likert scale (ranging from 1 = *Strongly disagree*, to 5 = *Strongly agree*). Higher summed scores indicate higher levels of the empathy characteristics specified. The psychometric properties of the BES were previously examined among Portuguese youth and demonstrated good results in terms of validity and reliability [26]. The internal consistency for the current study, estimated by α, was: Affective = .85, Cognitive = .81, and BES total = .77.

*Reactive-Proactive Aggression Questionnaire* (RPQ) [27]. This is a 23-item self-report measure of aggression that consists of two scales: reactive aggression (11 items; e.g., “Felt better after hitting or yelling at someone.”, “Hit others to defend yourself.”), and proactive aggression (12 items; e.g., “Carried a weapon to use in a fight.”, “Yelled at others so they would do things for you.”). Each item is scored on an ordinal 3-point Likert scale (ranging from 0 = *Never*, to 2 = *Often*). The items of each scale are summed to create each of the two scales scores, and a total RPQ score can also be used. Higher scores indicate higher levels of the aggression characteristics specified. The psychometric properties of the RPQ were previously examined among Portuguese youth and demonstrated good results in terms of validity and reliability [28]. The internal consistency for the current study, estimated by α, was: Reactive = .69, Proactive = .72, RPQ total = .75.

*Youth Psychopathic Traits Inventory–Triarchic–Short* (YPI-Tri-S) [29]. This is a 21-item brief measure of the Triarchic model of psychopathy that consists of three scales: Disinhibition (7 items; e.g., “It often happens that I do things without thinking ahead.”), Boldness (7 items; e.g., “What scares others usually doesn’t scare me.”), and Meanness (7 items; e.g., “I think that crying is a sign of weakness, even if no one sees you.”). Each item is scored on an ordinal 4-point Likert scale (ranging from 0 = *Does not apply at all*, to 3 = *Applies very well*). The items of each scale are summed to create each of the three scales scores, and a total score can also be used. Higher scores indicate higher levels of the characteristics specified. The psychometric properties of the YPI-Tri-S were previously examined among Portuguese youth and demonstrated good results in terms of validity and reliability [30]. The internal consistency for the current study, estimated by α, was: Boldness = .79, Disinhibition = .73, Meanness = .71, and YPI-Tri-S total = .81.

A sociodemographic questionnaire was constructed in order to describe the participants’ sociodemographic characteristics (e.g., nationality, gender, age, education). This questionnaire also included questions about alcohol, cannabis, and heroin/cocaine use (coded 0 = *Never*, 1 = *Sometimes*, and 2 = *Often*).

### Analyses

A series of Confirmatory Factor Analysis (CFA) were conducted using the EQS v6.4 software with correlation matrixes and Maximum Likelihood Robust (MLR) methods [31]. The following criteria were considered for an adequate fit: Comparative Fit Index (CFI) and Incremental Fit Index (IFI) > .90, Root Mean Square Error of Approximation (RMSEA 90% CI) < .08, and lowest Akaike Information Criterion (AIC); and for a good fit: CFI and IFI > .95, RMSEA 90% CI < .06, and lowest AIC. Satorra-Bentler chi-square/degrees of freedom (SBχ^2^/df) was also provided. The size of our sample was in line with the recommendations of at least a ratio of 10:1 (number of participants per number of items) when conducting CFA, and items with standardized loadings below .40 were excluded [32, 33]. Several possible models were examined: a one-factor model where all the items loaded on a single impulsivity latent factor; a model with inter-correlated two-factors where items loaded onto the two impulsivity factors: functional and dysfunctional; and a two-factor functional and dysfunctional impulsivity model with a second order higher factor. No modification indices were used to improve the fit of the different models.

We tested measurement invariance using the multigroup CFA modeling procedures described in the literature [34]. We first established a good-fitting baseline configural model, i.e., a valid model in women and men. Then, we tested the invariance of this model across groups of women and men by comparing the baseline configural model with constrained models to examine metric/weak invariance and scalar/strong invariance. Changes less than .01 in CFI (ΔCFI < .01), changes less than .015 in RMSEA (ΔRMSEA< .015), and non-significant SBχ^2^ difference tests were used as criteria for detecting invariance across groups [35].

SPSS Statistics v27 [36] was used to perform the additional classic psychometric analysis procedures. Correlations were considered low if < .20, high if >.50, and moderate if in between [37]; Fisher 2-tailed z-tests were used to analyze correlation strength differences; ANOVAs with effect size (partial Eta squared–η_*p*_^2^) were used to compare mean differences between groups [33]. Reliability was examined using item-total correlations (ITC; adequate if >.20), mean inter-item correlations (MIC; adequate if .15-.50 range), alpha and omega coefficients (adequate if >.70, good if >.80) [38].

## Results

### DII factor structure

Table 1 displays the different latent factor models tested and their respective goodness of fit indices. The two-factor model presented a better fit than the one-factor model and the two-factor second order model. However, some of the items showed standardized loadings below the recommended .40 cutoff, namely items 1 (“I don’t like to make decisions quickly, even simple decisions, such as choosing what to wear, or what to have for dinner.”, 11 (“I try to avoid activities where you have to act without much time to think first.”), and 13 (“I enjoy working out problems slowly and carefully.”). We decided to exclude those three items and run the model again. This modified model presented the best fit among the women and men samples and was subsequently used in the additional psychometric analysis procedures.

**Table 1:** Model fits of the DII.

	SBχ^2^/df	IFI	CFI	RMSEA (90% CI)	AIC
**Total sample**					
1-factor	9.69	.69	.69	.13(.14-.15)	1770.47
2-factor	2.93	.93	.93	.07(.06-.07)	213.96
2-factor i1 i11 i13 excluded	2.75	.95	.95	.06(.06-.07)	128.24
2-factor 2^nd^ order	2.45	.89	.89	.06(.05-.07)	76.41
**Men sample**					
1-factor	4.64	.71	.71	.13(.14-.15)	609.37
2-factor	1.95	.92	.92	.07(.06-.08)	-10.52
2-factor i1 i11 i13 excluded	1.80	.95	.94	.07(.05-.08)	-.32.78
2-factor 2^nd^ order	1.50	.90	.90	.06(.04-.07)	-67.28
**Woman sample**					
1-factor	5.09	.74	.74	.13(.12-.14)	711.85
2-factor	2.01	.94	.94	.07(.06-.07)	4.29
2-factor i1 i11 i13 excluded	2.14	.94	.94	.07(.06-.08)	24.97
2-factor 2^nd^ order	1.85	.88	.88	.06(.05-.07)	-23.10

*Note*. DII = Dickman Impulsivity Inventory; SBχ^2^ = Satorra-Bentler chi-square; df = degrees of freedom; IFI = Incremental Fit Index; CFI = Comparative Fit Index; RMSEA (90% CI) = Root Mean Square Error of Approximation (90% Confidence Interval); i1 i11 i13 excluded = items 1, 11, and 13 excluded.

Table 2 displays the item loadings for the 2-factor model after excluding items 1, 11 (from the functional impulsivity factor), and 13 (from the dysfunctional impulsivity factor) among the total, men and women samples. All items displayed loadings above the .40 recommended cutoff.

**Table 2:** Standardized loadings for the two-factor intercorrelated structure of the DII.

Items	Loadings
	T / M / W
**Functional Impulsivity**	
1. I don’t like to make decisions quickly, even simple decisions, such as choosing what to wear, or what to have for dinner. (R)	-- / -- / --
2. I am good at taking advantage of unexpected opportunities, where you have to do something immediately or lose your chance.	.56 /.59 / .52
3. Most of the time, I can put my thoughts into words very rapidly.	.47 / .48 / .47
4. I am uncomfortable when I have to make up my mind rapidly. (R)	.67 / .62 / .69
5. I like to take part in really fast-paced conversations, where you don’t have much time to think before you speak.	.53 / .58 / .52
6. I don’t like to do things quickly, even when I am doing something that is not very difficult. (R)	.43 / .43 / .40
7. I would enjoy working at a job that required me to make a lot of split-second decisions.	.63 /.61 / .63
8. I like sports and games in which you have to choose your next move very quickly.	.50 / .49 / .45
9. I have often missed out on opportunities because I couldn’t make up my mind fast enough. (R)	.73 / .72 / .73
10. People have admired me because I can think quickly.	.59 / .55 / .61
11. I try to avoid activities where you have to act without much time to think first. (R)	-- / -- / --
**Dysfunctional Impulsivity**	
12. I will often say whatever comes into my head without thinking first.	.48 /.42 / .55
13. I enjoy working out problems slowly and carefully. (R)	-- / -- / --
14. I frequently make appointments without thinking about whether I will be able to keep them.	.47 / .50 / .45
15. I frequently buy things without thinking about whether or not I can really afford them.	.42 /.41 / .43
16. I often make up my mind without taking the time to consider the situation from all angles.	.65 / .62 / .67
17. Often, I don’t spend enough time thinking over a situation before I act.	.61 / .55 / .67
18. I often get into trouble because I don’t think before I act.	.65 / .67 / .65
19. Many times the plans I make don’t work out because I haven’t gone over them carefully enough in advance.	.66 / .68 / .64
20. I rarely get involved in projects without first considering the potential problems. (R)	.70 / .70 / .71
21. Before making any important decision, I carefully weigh the pros and cons. (R)	.67 / .75 / .59
22. I am good at careful reasoning. (R)	.64 / .64 / .64
23. I often say and do things without considering the consequences.	.76 / .78 / .74

*Note*. DII = Dickman Impulsivity Inventory; T/M/W = Total/Men/Women samples; (R) = Reversible; -- / -- / -- = excluded.

Regarding gender measurement invariance, we first established the baseline configural model (SBχ^2^/df = 669.08/338, CFI = .94, RMSEA = .07). After that we tested metric invariance (factor loadings constrained across groups) with the results indicating a good fitting model (SBχ^2^/df = 688.53/356, ΔSBχ^2^/df = 22.25/18, *p* >.05, CFI = .94, ΔCFI = .00, RMSEA = .07, ΔRMSEA = .00). Finally, we tested scalar invariance (factor loadings and item intercepts constrained across groups) with the results indicating the presence of strong invariance (SBχ^2^/df = 691.68/359), ΔSBχ^2^/df = 45.36/21, *p* >.05, CFI = .94, ΔCFI = .00, RMSEA = .07, ΔRMSEA = .00). That is, the ΔCFIs were below the .01 cutoff, the ΔRMSEAs were below the .015 cutoff, and the ΔSBχ^2^ tests were non-significant. Such results indicated the presence of equivalence across the two groups.

### DII internal structure

The correlations between the two factors were non-significant in the men sample (*r* = .02, *p* >.05), but were positive and statistically significant in the women sample (*r* = .21, *p* < .01). These correlation values were statistically significant (*p* < .05) across gender using Fisher z-tests (2-tailed).

### DII external validity

Table 3 displays analyses examining the criterion-related validity of the DII. There were mostly positive, significant relationships between alcohol and cannabis drug use with the DII Dysfunctional factor but not with the DII Functional factor. There were mostly non-significant relationships with basic empathy in terms of the BES total and BES Affective factor. However, positive significant correlations were found between the BES Cognitive factor and the DII Functional factor, and negative significant correlations were found between the same BES Cognitive and the DII Dysfunctional factor. Lastly, there were mostly positive significant correlations between reactive-proactive aggression and the DII Dysfunctional factor, but not with the DII Functional factor. There were mostly positive, significant relationships between triarchic psychopathic traits and the DII Dysfunctional factor and the DII Functional factor.

**Table 3:** Criterion-related validity of the DII.

T/M/W	DII Functional	DII Dysfunctional
Alcohol	.08 / .03 / .10	.16** / .19** / .13
Cannabis	.09 / -.01 / .07	.25*** / .20** / .31***
Heroin/Cocaine	.10 / .11 / .07	.01 / .00 / -.00
BES total	-.01 / .06 / .05	-.04 / -.02 / -.04
BES Affective	-.15* / -.14 / -.05	.09 / .12 / .09
BES Cognitive	.16** / .26*** / .16*	-.18** / -.17* / -.18**
RPQ Total	.02 / -.04 / .08	.30*** / .28*** / .33***
RPQ Reactive	.00 / -.04 / .06	.25*** / .19** / .30***
RPQ Proactive	.06 / -.02 / .12	.31*** / .33*** / .28***
YPI-Tri-S total	.29*** / .23 ** / .25***	.42*** / .38*** / .46***
YPI-Tri-S Boldness	.33*** / .31*** / .28***	.09 / .05 / .14
YPI-Tri-S Disinhibition	.08 / .07 / .05	.58*** / .57*** / .59***
YPI-Tri-S Meanness	.22*** / -.10 / .24**	.24*** / .19** / .28***

*Note*. DII = Dickman Impulsivity Inventory; T/M/W = Total/Men/Women samples; BES = Basic Empathy Scale; RPQ = Reactive Proactive Aggression Questionnaire; YPI-Tri-S = Youth Psychopathic Traits Inventory–Triarchic–Short.

*** *p* < .001.

** *p* < .01.

* *p* < .05. Correlation values in bold indicate significant differences across gender

ANOVA results revealed significant differences between the groups of men and women regarding the Functional factor (*F* = 22.65, *p* < .001, η_p_^2^ = .05, *M*_men_ = 13.13, *SD*_men_ = 4.81, *M*_women_ = 10.98, *SD*_women_ = 4.51), but not regarding the Dysfunctional factor (*F* = .61, *p* = .44, η_p_^2^ = .00, *M*_men_ = 7.82, *SD*_men_ = 5.66, *M*_women_ = 7.41, *SD*_women_ = 5.29).

### DII internal consistency

Table 4 presents the internal consistency of the DII estimated using several methods, namely ITC, MIC, alpha and omega coefficients. These values can be considered mostly good in both the men and women samples.

**Table 4:** Internal consistency of the DII.

M/W	DII Functional	DII Dysfunctional
ITC	.38-.63 / .36-.64	.38-.69 /.41-.69
MIC	.32 / .31	.37 / .38
Alpha	.81 / .80	.87 / .87
Omega	.82 / .83	.89 / .88

*Note*. DII = Dickman Impulsivity Inventory; M/W = Men/Women samples; ITC = Item-total correlation ranges; MIC = Mean inter-item correlations.

## Discussion

The present investigation was the first to examine the DII among university students in Portugal. CFAs showed that the 2-factor first-order model that excluded items 1, 11, and 13 (due to low standardized loadings) presented the best fit when compared to the other models we tested (e.g., the 1-factor model). Previous studies examining validity of the DII conducted in other countries [12, 14] obtained support for a 2-factor model based on culturally distinct samples after removing specific items that were performing poorly in terms of standardized loadings. However, it is worth mentioning that none of the specific items removed in the present study were considered problematic in previous studies examining validity of the DII that we are aware of. Considering that there is always a tradeoff between removing items and retaining the measure as originally designed, we think that the removal of the three items is the preferred approach as it results in better psychometric properties and did not significantly affect the representativity of the DII item pool because the removed items were spread relatively evenly across the two subscales. The 2-factor 2^nd^ order reached an acceptable fit among men but not women. Since only 2^nd^ order models with good fits justify the use of a total score, we decided not to use a total score with our Portuguese validation of the DII. This is consistent with all the existent validations of the DII that also did not consider a total score.

Next, we examined measurement invariance across gender. Results supported the presence of weak and strong invariance with the modified 2-factor first-order model. Such invariance results indicate that this model is sharing an appropriate level of equivalence across gender that justifies unbiased group mean comparisons. Our investigation is one of the few studies that has examined measurement invariance of the DII, and we replicated previous findings that supported factor similarities between gender and gender invariance [9, 12]. Establishing invariance is fundamental to justify group comparison (e.g., across gender, across age). For example, establishing measurement invariance of the DII in a Portuguese sample allows for the examination of Caci et al.’s [9] suggestion that the Dysfunctional factor may be operating differently across gender. The correlations between the Functional and Dysfunctional factors of the DII across gender were low and non-significant for the sample of men, but moderate and significant for the sample of women. Although previous studies reported similar results both in terms of low and non-significant correlations [10, 14] and moderate to low and significant correlations [5, 9, 11, 13], such differences could be attributed to invariance problems in studies that did not first determine that the DII was assessing impulsivity the same across men and women. Our findings suggest such differences are not driven by measurement invariance problems. Even more so, our findings are more unusual because the correlations were significantly different across gender, i.e., statistically stronger among women than among men. This suggests that among women there is a larger overlap between the Functional and Dysfunctional factors of the DII than there is for men, a finding that was also reported by Colledani [12].

Evidence for the criterion-related validity of the DII factors with alcohol, cannabis, and heroin/cocaine use mostly revealed correlations of the Dysfunctional factor with the first two substances (with the exception of alcohol use among women). No significant correlations emerged with heroin/cocaine use, which is most likely a result of the low levels of heroin/cocaine use among university students. As expected, the Functional factor showed no significant correlations with substance use. Evidence for criterion-related validity was also examined with the basic empathy measure showed mostly the expected non-significant correlations, specifically with the empathy total and with emotional empathy. It also showed positive correlations of the Functional factor with cognitive empathy, and negative correlations of the Dysfunctional factor with cognitive empathy among both men and women. That is, cognitive empathy is positively associated with functional impulsivity effective outcomes and negatively associated with dysfunctional impulsivity ineffective outcomes. A possible explanation for these findings is that dysfunctional impulsive behaviors may hinder appropriate rational actions based on cognitive empathic skills [39].

Evidence for the criterion-related validity of the DII factors was also examined with measures of aggression and triarchic psychopathic traits. Positive moderate to low significant correlations were found only between the Dysfunctional factor and aggression (reactive, proactive and total aggression) among men and women. That is, aggression, either reactive or proactive, is only associated with dysfunctional impulsivity ineffective outcomes. Evidence for the criterion-related validity with triarchic psychopathic traits showed mostly positive moderate significant correlations. The Functional factor was mostly associated with Boldness, which is usually described as the triarchic factor with the better overtone. Bold individuals are described as dashing, assertive, and have a dominant interpersonal style and sense of adventure. They are self-assured, recover quickly from stressful situations and have a tolerance for unfamiliarity and danger. It is important to mention that Boldness relates to social functioning and is a general population feature. It is not limited to antisocial or criminal individuals. The Dysfunctional factor was mostly associated with the Disinhibition and Meanness factors of the YPI-Tri-S, which are the triarchic factors with the worse overtone. Disinhibited individuals are described as impulsive, irresponsible, and as having self-control deficits in a variety of contexts. Meanness is characterized by a defiance of authority (e.g., parents, police, teachers), verbal derisiveness, physical cruelty, and disparate forms of aggression, destructiveness, and the targeted exploitation of people for gain [40, 41]. Also, the correlations between the Functional factor and the Meanness factor of the YPI-Tri-S were only significant for women, suggesting that functional impulsive women tend to have a mean streak.

In terms of the known-groups validity, the comparisons of men and women participants revealed that men obtained significantly higher scores on the Functional factor but not on the Dysfunctional factor. Most of the literature recognizes that men tend to be more impulsive on impulsivity measures when compared to women [15], including the DII [9, 10, 12] and the Barratt Impulsiveness Scale version 11 (BIS-11) [42, 43]. However, at least one previous study examining the DII [12] also showed significantly higher functional impulsivity, but not dysfunctional impulsivity, in men. In this regard, the findings of our study are somewhat mixed. Again, our findings support such differences between men and women and suggest these differences are not caused by measurement invariance problems.

The reliability values for the DII indicated good reliability in both the men and women samples with the values being very similar across gender. The Functional and Dysfunctional factors of the DII showed good values in terms of the item-total correlations (i.e., above .30), mean inter-item correlations (i.e., within the .15-.50 range), Cronbach’s alpha and Omega coefficient (i.e., above .80). The Cronbach’s alphas for the DII Functional and Dysfunctional factors of our study were similar to the ones obtained in the Dutch version (.84 and .85) [10], and better than the ones obtained in the Brazilian (.73 and .75) [14], North-American (.74 and .85) [5], Italian (.75 and .78) [12], French (.75 and .79) [9], Spanish (.78 and .76) [11], and Chinese (.68 and .75) [13] versions, respectively.

We must point out some limitations of our study. Our sample was a convenience sample of college students that cannot be considered representative of the whole Portuguese population. Because our sample originated from a college it may not include very high-risk youth (e.g., at risk of delinquency, at risk of drug addiction) and our findings may not generalize to such populations. We relied exclusively on self-report methodology, which can cause common method bias and affect the ability and openness of the participants to respond honestly. Another limitation was caused by our removal of some DII items that were not functioning properly in our Portuguese sample. This problem, however, was also present in other validations of this measure.

## Conclusions

We conclude that the Portuguese version of the DII presents evidence of good psychometric properties in terms of validity and reliability among our sample of men and women college students, including weak and strong measurement invariance that legitimates comparisons across gender. The availability of a brief, easy-to-use measure of functional and dysfunctional behaviors can be important for identifying and intervening in maladaptive risk-taking in youth. Testing of the Portuguese version of this important instrument is still ongoing, and additional psychometric procedures (e.g., test-retest reliability, cross-validation) should be conducted in the near future. We hope the present work catalyzes future studies on functional and dysfunctional impulsivity in this population.

## Supporting information

S1 Appendix Original and Portuguese translation of the DII. (DOCX)

S1 Data (SAV)
