# Supplementary material for: The Dickman Impulsivity Inventory: Validation and measurement invariance among Portuguese young adults
Source: PLoS One. 2021 Dec 2;16(12):e0260621. doi: 10.1371/journal.pone.0260621 (PMC8638866; doi:10.1371/journal.pone.0260621)
Supplement: S1 Appendix — (DOCX) [file pone.0260621.s001.docx]

**Appendix 1. Original and Portuguese translation of the DII.**

| Original | Portuguese translation |
| --- | --- |
| **Functional Impulsivity**  1. I don't like to make decisions quickly, even simple decisions, such as choosing what to wear, or what to have for dinner. (R)  2. I am good at taking advantage of unexpected opportunities, where you have to do something immediately or lose your chance.  3. Most of the time, I can put my thoughts into words very rapidly.  4. I am uncomfortable when I have to make up my mind rapidly. (R)  5. I like to take part in really fast-paced conversations, where you don't have much time to think before you speak.  6. I don't like to do things quickly, even when I am doing something that is not very difficult. (R)  7. I would enjoy working at a job that required me to make a lot of split-second decisions.  8. I like sports and games in which you have to choose your next move very quickly.  9. I have often missed out on opportunities because I couldn't make up my mind fast enough. (R)  10. People have admired me because I can think quickly.  11. I try to avoid activities where you have to act without much time to think first. (R)  **Dysfunctional Impulsivity**  12. I will often say whatever comes into my head without thinking first.  13. I enjoy working out problems slowly and carefully. (R)  14. I frequently make appointments without thinking about whether I will be able to keep them.  15. I frequently buy things without thinking about whether or not I can really afford them.  16. I often make up my mind without taking the time to consider the situation from all angles.  17. Often, I don't spend enough time thinking over a situation before I act.  18. I often get into trouble because I don't think before I act.  19. Many times the plans I make don’t work out because I haven’t gone over them carefully enough in advance.  20. I rarely get involved in projects without first considering the potential problems. (R)  21. Before making any important decision, I carefully weigh the pros and cons. (R)  22. I am good at careful reasoning. (R)  23. I often say and do things without considering the consequences. | **Impulsividade Funcional**  1. Não gosto de tomar decisões rapidamente, mesmo que sejam decisões simples tais como escolher o que vestir ou o que comer ao jantar. (R)  2. Sou bom a tirar vantagem de oportunidades inesperadas em que se tem de agir imediatamente ou perder a oportunidade.  3. Na maior parte do tempo, sou capaz de colocar os meus pensamentos em palavras muito rapidamente.  4. Sinto-me desconfortável quando tenho de me decidir rapidamente. (R)  5. Gosto de participar em conversas com ritmo muito acelerado onde não se tem muito tempo para pensar antes de se falar.  6. Não gosto de fazer as coisas rapidamente mesmo quando estou a fazer algo que não é muito difícil. (R)  7. Gostaria de trabalhar num emprego em que se tem de tomar decisões rapidamente em frações de segundo.  8. Gosto de desportos e jogos nos quais se tem de decidir o próximo passo rapidamente.  9. Frequentemente perdi oportunidades porque não me consegui decidir rapidamente o suficiente. (R)  10. As pessoas admiram-me porque consigo pensar rapidamente.  11. Tento evitar atividades onde tenho de agir sem ter muito tempo para pensar primeiro. (R)  **Impulsividade Disfuncional**  12. Frequentemente digo o que me vem à cabeça sem pensar primeiro.  13. Eu gosto de resolver problemas de forma lenta e cuidadosa. (R)  14. Frequentemente marco compromissos sem pensar se consigo cumpri-los ou não.  15. Frequentemente compro coisas sem pensar bem se as posso pagar ou não.  16. Muitas vezes decido-me sem refletir o tempo necessário para ver a situação de várias perspetivas.  17. Frequentemente não gasto tempo a pensar numa situação antes de agir.  18. Frequentemente meto-me em problemas porque não penso antes de agir.  19. Muitas vezes os planos que faço não funcionam porque não pensei neles cuidadosamente com antecedência.  20. Raramente envolvo-me em projetos sem primeiro considerar os potenciais problemas. (R)  21. Antes de tomar quaisquer decisões importantes, penso cuidadosamente nos prós e contras. (R)  22. Eu sou bom a raciocinar cuidadosamente. (R)  23. Frequentemente digo e faço coisas sem considerar as consequências. |

*Note*. DII = Dickman Impulsivity Inventory
